# Tweet Content Related to Sexually Transmitted Diseases: No Joking Matter

**DOI:** 10.2196/jmir.3259

**Published:** 2014-10-06

**Authors:** Elia Gabarron, J Artur Serrano, Rolf Wynn, Annie YS Lau

**Affiliations:** ^1^NST-Norwegian Centre for Integrated Care and TelemedicineUniversity Hospital North NorwayTromsøNorway; ^2^Department of Clinical MedicineFaculty of Health SciencesThe Arctic University of NorwayTromsøNorway; ^3^Division of Addictions and Specialized Psychiatric ServicesUniversity Hospital North NorwayTromsøNorway; ^4^Centre for Health InformaticsAustralian Institute of Health InnovationUniversity of New South WalesSydneyAustralia

**Keywords:** Internet, chlamydia, HIV, Twitter messaging

## Abstract

**Background:**

Online social media, such as the microblogging site Twitter, have become a space for speedy exchange of information regarding sexually transmitted diseases (STDs), presenting a potential risk environment for how STDs are portrayed. Examining the types of “tweeters” (users who post messages on Twitter) and the nature of “tweet” messages is important for identifying how information related to STDs is posted in online social media.

**Objective:**

The intent of the study was to describe the types of message emitters on Twitter in relation to two different STDs—chlamydia and human immunodeficiency virus (HIV)—as well as the nature of content tweeted, including how seriously the topic was treated.

**Methods:**

We used the Twitter search engine to look for tweets posted worldwide from August 1-7, 2013, and from September 1-7, 2013, containing the words “chlamydia” or “HIV”, and the hashtags “#chlamydia” or “#HIV”. Tweeters were classified by two independent reviewers according to the type of avatar of the user (human, logo, or fantasy), the identification of the emitter (identifiable, semi-identifiable, or non-identifiable), and the source (private company, general media, scientific media, non-governmental, individual account, academic institution, government department, or undefined). Tweet messages were also independently classified according to their nature (serious or jokes/funny), and whether their main message was factual or of a personal nature/experience.

**Results:**

A total of 694 tweets were posted by 426 different users during the first 7 days of August and September, containing the hashtags and/or simple words “chlamydia” and/or “HIV”. Jokes or funny tweets were more frequently posted by individual users (89%, 66/74), with a human avatar (81%, 60/74), from a non-identifiable user (72%, 53/74), and they were most frequently related to chlamydia (76%, 56/74). Serious tweets were most frequently posted by the general media (20.6%, 128/620), using a logo avatar (66.9%, 415/620), and with identifiable accounts (85.2%, 528/620). No government departments, non-governmental organizations, scientific media, or academic institutions posted a joke on STDs. A total of 104 of these analyzed tweets were re-tweeted messages, belonging to 68 unique tweets. The content was serious (99%, 67/68), factual (90%, 52/58), and about HIV (85%, 58/68).

**Conclusions:**

Social media such as Twitter may be an important source of information regarding STDs provided that the topic is presented appropriately. Reassuringly, the study showed that almost 9/10 of tweets on STDs (chlamydia and HIV) were of serious content, and many of the tweets that were re-tweeted were facts. The jokes that were tweeted were mainly about chlamydia, and posted by non-identifiable emitters. We believe social media should be used to an even larger extent to disseminate correct information about STDs.

## Introduction

In recent years, the Internet has changed the way people access general health information and make decisions about their health care [1]. The Internet has become the leading source for seeking information regarding sensitive topics, such as sexual health [2] or sexually transmitted diseases (STDs) [3].

The use of the Internet and online social media in relation to sexual health and STDs has been studied recently [4-6]. Some studies have shown that the Internet and online social media can represent a new “risk environment” in which distorted, wrong, and stigmatizing information can be published and spread rapidly [7-9], can be a space where potentially STD-infected sex partners can meet [5,10,11], and also represent a space for posting and sharing unhealthy attitudes. An example of this can be found in a study carried out on the teen dating website “Mylol.net”, showing that among adolescents’ self-presentation, 27.7% displayed risky behavior, and 15.8% risky sexual behavior [12], with the potential effect of attracting unwanted attention from cyberbullies or sexual predators [13].

But the fact that the Internet, and specifically online social networks, are accessible to an increasing number of people, allows these powerful media tools to disseminate and inform users about evidence-based material on health [14], including sensitive topics such as sexual health and STDs [15]. Online social media are also valued as environments for their potential to engage with the general public [16,17], and particularly young people [18]. In particular, evidence suggests that certain health behaviors and sexual health behaviors might spread through social ties, of which online social networks are one example [19,20]. Recently, online social networking sites, such as Facebook or the microblogging site Twitter, have started to be used for sexual health promotion and sexual health education [10]. These online social networks may be regarded as a promising and new field for educating people about STDs. 

Despite the assumed potential benefits of using online social media to promote and disseminate information on healthy sexual behaviors, there is a lack of understanding regarding how they are being used in relation to STDs. The aim of this study is to describe the nature of message emitters in the online social network Twitter on two different sexually transmitted diseases—chlamydia and human immunodeficiency virus (HIV)—the content of their tweets, and the prevalence of tweets related to unhealthy sexual health behaviors and attitudes.

## Methods

### Search and Data Extraction

We used the Twitter search engine to look for tweets posted worldwide in this social media, from August 1-7, 2013 and from September 1-7, 2013, containing the words “chlamydia” or “HIV”, and the hashtags “#chlamydia” or “#HIV”. Twitter was selected for being one of the fastest growing social media platforms, with roughly 500 million tweets posted every day, and where all the posted information is fully available, even for people who have not created a Twitter account [21]. We selected these two STDs (chlamydia and HIV) because they have a common transmission mode, but with vastly different prognoses and outcomes. At the time of the study, there were two options available on the search engine to retrieve posted tweets: via keywords or via people. The search using keywords gives the option of having an overview of the people who posted in this area and the contexts in which these keywords were being used. Searches via hashtags were also conducted as they provide specific conversations focusing on these topics; hashtags are commonly used on Twitter to connect people who share a similar dialogue, and they are also used by individuals and agencies to filter tweets on a specific topic.

In regard to the months selected for examining these tweet messages, we selected August, which could be considered a common holiday month in the northern hemisphere, where the majority of Twitter users are located [22]. We chose a holiday month because people on holiday have more free time to read and post tweets and previous research has suggested there might be a higher likelihood of high-risk sexual behavior during holidays [23]. In addition, we chose September as an example of a working month.

We extracted the date of all the retrieved tweets (including both unique tweets and re-tweets), the name and avatar of emitters, and the text of the posted tweets in our analysis.

### Code Categories

Emitters’ profile information (source of the emitter) and their posted messages (tone and nature), of all the downloaded tweets in English, were classified categorically by two independent reviewers (AS and EG). Any discrepancies regarding the categorization of the tweets were discussed with a third additional independent reviewer (AL) until consensus was reached. The inter-rater agreement was obtained for the three categories. A 95% confidence interval was found using the generic formula for 95% confidence intervals (estimate ± SE 1.96). Chi-square tests were used for categorical variables. All data were analyzed with SPSS version 19 for Mac.

Tweet emitters, also called “tweeters”, were classified according to:

- Type of avatar (via image): whether the image contained at least a person, a logo image, or a fantasy image. In cases where two or more of these images were displayed at the same time in the avatar, the human image was considered. If the logo image and the fantasy image were displayed at same time in the avatar, the logo image was considered.
- Identification of the emitter (via textual profile information): identifiable (ie, the full name of the person or entity that was tweeting was clearly present), semi-identifiable (ie, identification of who was tweeting was not clearly exposed), or non-identifiable (ie, not possible to identify the person or entity responsible for the tweets).
- Source of the emitter (via textual profile information): private company, general media (newspapers, magazines, TV, and radio), scientific media (mostly scientific journals), a non-governmental organization, an individual account, an academic institution (university, college), a government department, or undefined (accounts that could not be classified in any of the previous categories).

Tweet messages were also independently classified according to:

- Tone (serious or jokes/funny): tweets were classified as jokes, according to the dictionary definition of a joke as “a thing that someone says to cause amusement or laughter, especially a story with a funny punchline” [24]. The remaining, which could not be classified as a jokes, were considered serious tweets.
- Nature: whether the main message was factual (a general topic, not related to an individual experience), or of a personal nature/experience.

## Results

**Figure 1:**
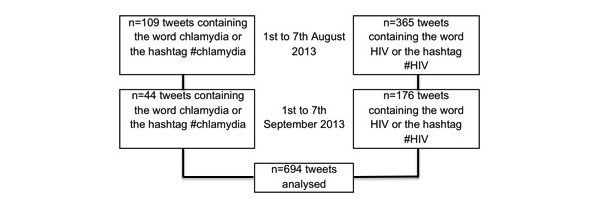
Search and study selection process of tweets about chlamydia and HIV on Twitter.

A total of 694 tweets (of which 104 were re-tweets) containing the hashtags and/or simple words “chlamydia” and/or “HIV”, were posted by 426 different users during the first 7 days of August and the first 7 days of September 2013. Search results are summarized in Figure 1. Of those, 332 different users posted 541 tweets on HIV, and 79 different users posted 153 tweets on chlamydia. Regarding the type of avatar or image used by those 426 primary case users, 220 (51.6%) of them showed logos, 162 (38.0%) a human image, and 44 (10.3%) a fantasy avatar. In 324 cases (76.1%), the user account was considered identifiable, in 6 (1.4%) semi-identifiable, and in 96 (22.5%) non-identifiable.

The 694 tweets were classified as tweeted by individual users in 231 (33.3%) cases, the general media in 132 (19.0%) tweets, a government department in 114 (16.4%) tweets, a non-governmental organization in 90 (13.0%) tweets, scientific media in 55 (7.9%) tweets, a private company in 19 (2.7%) tweets, an academic institution in 12 (1.7%) tweets, and the nature of the emitter was undefined in 41 (5.9%) tweets.

A total of 695 tweets were downloaded, but one tweet was removed from the analysis because, although it included the word “HIV”, it was a text message written in Gaelic language and not related to STDs. Thus, the total number of tweets analyzed was 694. Regarding the categorization of the tweet messages as jokes/serious, only 14 discrepancies were found between reviewers, and the kappa value was found to be .893, almost perfect agreement, according to Landis and Koch [25]. In terms of the nature of tweets (personal experience or fact), 83 discrepancies were found in the first review round, with an inter-rater agreement of kappa=.667, considered as a substantial agreement [25]. Regarding the source of information, 145 discrepancies were found between the two reviewers, and the kappa value was .729, which represents a substantial agreement [25].

Most of the tweets posted on STDs were considered to be serious (89.3%, 620/694); however, some jokes or funny messages were found on Twitter (10.7%, 74/694), they were most frequently posted by individual users (89%, 66/74), with a human avatar (81%, 60/74) and posted by non-identifiable users (72%, 53/74). On the other side, serious tweets were most frequently posted by news organizations (20.6%, 128/620), using a logo avatar (66.9%, 415/620), and classified as identifiable accounts (85.2%, 528/620). Jokes in tweet messages were frequently related to chlamydia (76%, 56/74). No tweets of a joking nature were posted by government departments, non-governmental organizations, scientific media, or academic institutions. Tweets that contain jokes related to STDs typically convey a highly inappropriate and misguided view toward the disease, for example: “If it weren’t an STD, I would consider naming my daughter Chlamydia #pretty”, or “What’s the most positive thing in Africa?? HIV”.

**Figure 2:**
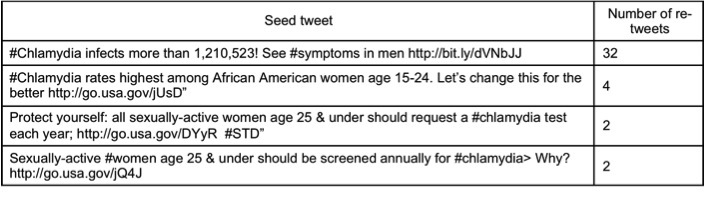
Examples of messages that have been re-tweeted more than once.

Table 1 describes the features of tweeters according to the nature and the tone of their tweets: funny/jokes or serious, and fact or personal experience. Of the total number of analyzed tweets, 104 were re-tweeted messages. These 104 re-tweets correspond to 68 unique tweets (seed tweets), where 4 messages were re-tweeted more than once (Figure 2). The features of the user account and content of these 68 seed tweets are summarized in Table 2.

**Table 1:** Tone and nature of the tweets.

		Joke / Fun	Serious	Fact	Personal experience	Total
		n=74 (10.7%)	n=620 (89.3%)	n=555 (80.0%)	n=139 (20.0%)	N=694 (100%)
**User image (avatar)** ^a^						
	Human	60 (81.1%)	134 (21.6%)	98 (17.7%)	96 (69.1%)	194 (28.0%)
	Fantasy	9 (12.2%)	71 (11.5%)	66 (11.9%)	14 (10.1%)	80 (11.5%)
	Logo	5 (6.8%)	415 (66.9%)	391 (70.5%)	29 (20.9%)	420 (60.5%)
**Type of account user** ^a^						
	Identifiable	21 (28.4%)	528 (85.2%)	483 (87.0%)	66 (47.5%)	549 (79.1%)
	Semi-identifiable	0 (0%)	15 (2.4%)	13 (2.3%)	2 (1.4%)	15 (2.2%)
	Non-identifiable	53 (71.6%)	77 (12.4%)	59 (10.6%)	71 (51.1%)	130 (18.7%)
**Tweet emitter** ^a^						
	Individual	66 (89.2%)	165 (26.6%)	125 (22.5%)	106 (76.3%)	231 (33.3%)
	General media	4 (5.4%)	128 (20.6%)	118 (21.3%)	14 (10.1%)	187 (26.9%)
	Scientific media	0 (0%)	55 (8.9%)	55 (9.9%)	0 (0%)	12 (1.7%)
	Government department	0 (0%)	12 (1.9%)	111 (20.0%)	3 (2.2%)	90 (13.0%)
	Non-governmental	0 (0%)	90 (14.5%)	81 (14.6%)	9 (6.5%)	41 (5.9%)
	Undefined	3 (4.1%)	38 (6.1%)	35 (6.3%)	6 (4.3%)	19 (2.7%)
	Private company	1 (1.4%)	18 (2.9%)	18 (3.2%)	1 (0.7%)	12 (1.7%)
	Academic institution	0 (0%)	12 (1.9%)	12 (2.2%)	0 (0%)	102 (14.7%)
**STD** ^a^						
	HIV	18 (24.3%)	523 (84.4%)	461 (83.1%)	80 (57.6%)	541 (78.0%)
	Chlamydia	56 (75.7%)	97 (15.6%)	94 (16.9%)	59 (42.4%)	153 (22.0%)
**STD search** ^a^						
	Word HIV	18 (24.3%)	451 (72.7%)	395 (71.2%)	74 (53.2%)	469 (67.6%)
	Hashtag #HIV	0 (0%)	72 (11.6%)	66 (11.9%)	6 (4.3%)	72 (10.4%)
	Word chlamydia	41 (55.4%)	10 (1.6%)	8 (1.4%)	43 (90.9%)	51 (7.3%)
	Hashtag #chlamydia	15 (20.3%)	87 (14.0%)	86 (15.5%)	16 (11.5%)	102 (14.7%)

^a^Chi-square, *P*<.001

**Table 2:** Features of the 68 re-tweeted messages.

Feature		Chlamydia n=10 (15%)	HIV n=58 (85%)
**User image** ^a^			
	Logo	1 (10%)	0 (0%)
	Human	1 (10%)	8 (14%)
	Fantasy	8 (80%)	50 (86%)
**Type of account user** ^a^			
	Identifiable	9 (90%)	56 (97%)
	Semi-identifiable	0 (0%)	0 (0%)
	Non-identifiable	1 (10%)	2 (3%)
**Tweet emitter** ^a^			
	Individual	1 (10%)	8 (14%)
	General media	2 (20%)	11 (19%)
	Scientific media	1 (10%)	8 (14%)
	Government department	6 (60%)	12 (21%)
	Non-governmental	0 (0%)	15 (26%)
	Undefined	0 (0%)	1 (2%)
	Private company	0 (0%)	3 (5%)
	Academic institution	0 (0%)	0 (0%)
**Tone of the message** ^a^			
	Joke / Funny	1 (10%)	0 (0%)
	Serious	9 (90%)	58 (100%)
**Nature of the message** ^a^			
	Fact	9 (90%)	52 (90%)
	Personal experience	1 (10%)	6 (10%)

^a^Chi-square, *P*<.001

## Discussion

### Principal Findings

In our analysis of tweets on STDs (ie, HIV or chlamydia) posted during the first 7 days of August and September 2013, we found that nearly 9 of 10 tweets focused on HIV (620 of the total 694). This higher frequency of tweets about HIV may be explained by an increased awareness of the disease, to the detriment of chlamydia, which may be a reflection of what happens in society.

Regarding the month of the posts, almost 7 of 10 were tweeted during August (474 of the total of 694), which could be considered a holiday month. Holiday months can be seen as periods in which people have more free time to read and post tweets, but also periods in which people may have a higher rate of sexual activity, and likelihood of higher-risk sexual behavior [23]. Posting health promotion messages on Twitter during a holiday month (eg, Christmas) could potentially represent an educational or even a preventive tool in a period when there is an increased likelihood of higher-risk sexual behavior.

Some jokes or funny remarks on STDs were also posted. They were more frequently tweeted by individual users, with a human avatar, and came from a non-identifiable user (ie, very difficult to ascertain their real identity). The jokes were most frequently posted on chlamydia.

On the other hand, serious tweets on STDs were posted more frequently by users using a logo as avatar, had easily identifiable emitters, and belonged more frequently to news organizations, scientific media, and individuals. Almost all the re-tweeted messages were of serious content as well, and in these cases, the first tweet in the sequence belonged to an identifiable user too, using a fantasy avatar, and were mostly from the general media, the scientific media, government agencies, and non-governmental organizations. Regarding the messages re-tweeted, it is also interesting to stress that although most of the re-tweets were on HIV, the four messages that were re-tweeted two or more times were on chlamydia.

Tweets on personal experiences with STDs were posted more frequently by individual users, with a human avatar, and they included the word chlamydia in their tweets. Personal experience tweets were longer than tweets of a factual nature. In addition, tweets on STD-related facts were posted more frequently by identifiable users with a logo image, and had a higher frequency of re-tweets.

Overall, sexually transmitted diseases are a worrying problem worldwide. While both diseases are sexually transmitted, HIV and chlamydia appear to be seen as very different issues in online social media. While information related to HIV stresses the disease as a serious topic (such as treatment, prevention, or stigma), chlamydia is more frequently related to joke tweets.

### Behavior and Seriousness of Tweets on STDs

While online and social media communication may share many features with face-to-face communication, there are important differences [26]. Some differences may be most pronounced when the communication is anonymous. For example, self-disclosure [27] and disinhibition [28] are found to be more frequent in online communication, such as social media, where people have the option of camouflaging themselves when talking about sensitive topics, than in face-to-face mode. Anonymous communication might be beneficial for some people, for instance people who suffer from HIV or other STDs may discuss their experiences or concerns if allowed to do so anonymously. On the other hand, anonymity may be exploited negatively, for instance to make offensive statements or to deceive someone.

Our findings can be understood in at least two different ways. First, one may postulate that the majority of chlamydia joking-related messages stems primarily from anonymous tweeters [28]. In this respect, the joking behavior may be understood as resulting from the lack of social norms or group values contingent on tweeters’ anonymity, where the ability to hide behind an anonymous avatar on Twitter allows one to adopt a disinhibited behavior with no responsibility or accountability.

Second, one may also see the differential treatment given to chlamydia and HIV as a result of group norms, suggesting it is acceptable to tweet chlamydia-related jokes but that tweeting HIV-related jokes is not acceptable. Following this reasoning, in situations when one can get away with anonymity, such as computer-mediated communication, the Social Identity Model of Deindividuation Effects (SIDE) [29] suggests that group norms may become even more influential, and group members tend to follow behaviors set by these existing social norms even when these behaviors are deemed unacceptable in non-anonymous settings [30,31].

This reasoning suggests that in the Twitter community, tweets related to jokes on chlamydia are more likely to be acceptable (although most who do so prefer to do it anonymously), while jokes pertaining to HIV are unacceptable. The reason why such a distinction is made is unclear, but it may be related to the severity of the disease (ie, HIV is potentially deadly, chlamydia is not), or concerns regarding stigmatization (ie, the HIV epidemic has affected some groups of the population more than others), or other factors we are unaware of. While it is encouraging to see that only factual information or non-joking material is being re-tweeted at the time of analysis, many jokes can still be found on Twitter.

### Twitter as a Channel for Sensitive Information

It is a fact that chlamydia is a less severe disease than HIV, and that if chlamydia is diagnosed early, it can easily be cured [32]. However, few people do the tests to check if they have chlamydia, and in many cases the disease may become chronic [32]. Both infections, HIV and chlamydia (and other STDs), can be spread by exactly the same sexual risk-taking behaviors [32]. We believe social media could give hard-to-reach young people unique access to high quality information about the sensitive topic of STDs. Some governmental and health organizations are already exchanging information about STDs on Twitter, such as USAID Education and the Centers for Disease Control and Prevention. However, the topic of their tweets on STDs is mainly HIV. This is possibly due to the fact that from an organizational perspective, there are agencies that have a primary mandate or focus on HIV (such as AIDS service organizations), but few have a focus specifically on chlamydia (being much more likely to have a focus on all STDs or on sexual health more broadly). Other stakeholders should pay attention to the discussion in online social media and could utilize this channel more frequently in the preventive work against STDs.

As in other online social media and on the Internet in general, consumers can find distorted information on Twitter [7,8,33,34]. Currently, there is no shortage of jokes, funny remarks, and other comments on STDs on Twitter. Although we did not classify tweets for its misguided content, some of the tweets may communicate a misguided attitude on STDs (eg, where having an STD is something to be proud of), which is not in line with the important task of reducing the spread of STDs.

Preventing and containing the spread of false information and myths on STDs should be an important item on any public health agenda. In particular, content on Twitter and other online social media outlets is available to a large number of consumers, where misguided information can spread rapidly [7-9]. Myths and misinformed attitudes that are not contained can potentially lead to dire consequences on our health attitudes, decisions, and actions [8,9,30]. Strategies for creating social awareness regarding the spread of misinformation and for finding ways to improve the quality of the information should be addressed with careful planning.

Government departments, non-governmental organizations, and academic institutions represent a more trustworthy source of information on STDs. In fact, 18% of the tweets came from government agencies (government departments and academic institutions), and 16% from non-governmental organizations, which is not a bad start but is an area that could be improved. These agencies should consider increasing their presence and visibility on social media in order to reach their target groups with high quality information, and to counteract some of the more flippant tweets that downplay the health risks of STDs.

In future research, it would be of interest to analyze other online social media, such as Facebook or YouTube, in order to examine how the topic of STDs is presented in these platforms.

### Limitations

The study has several limitations. We selected two STDs with a common transmission mode, but with vastly different prognoses and outcomes (chlamydia and HIV). For the search conducted on Twitter, we only focused on tweets using the word or the hashtag “chlamydia” or “HIV”, which may have reduced the number of tweets related to the topic. Future studies should broaden the scope of keywords and hashtags, for example, including terms such as “AIDS”.

Although the search was conducted worldwide, the terms used implied that mostly tweets in English were identified.

Taking into account that we analyzed only two STDs on Twitter (HIV, chlamydia), and we used only English words, our results cannot be generalized to other online social media platforms or to other settings where English is not the primary language.

The number of Twitter followers associated with users posting on STDs was not collected. Future research should consider collecting that information, and investigate the spread rate of tweets.

### Conclusions

The study showed that nearly 9 of every 10 tweets on STDs (chlamydia and HIV) were of serious content, and many of the tweets that were re-tweeted were facts. We believe this finding is reassuring as it suggests that most content on Twitter relating to STDs is of a factual and serious nature, which we hope might help in informing people about these diseases. However, many jokes could also be found, mainly about chlamydia, and these jokes and funny remarks were typically posted by non-identifiable emitters. For social media such as Twitter to be considered an important source of public health information regarding STDs, the topic needs to be presented appropriately. We believe social media plays an important role in the next generation of public health tools in disseminating correct information about STDs.

## Abbreviations

HIV: human immunodeficiency virus
STD: sexually transmitted disease
